# Comparatively analysing the postoperative optical performance of different intraocular lenses: a prospective observational study

**DOI:** 10.1186/s12886-024-03439-0

**Published:** 2024-04-26

**Authors:** Shuanglin Guo, Hao Huang, Bowen Li, Mansha Huang, Lu Gao, Jingyi Chen, Yuying Zeng, Ye Yang, Lin Liu, Lu Cheng, Siyang Yao, Hao Cheng

**Affiliations:** 1 Department of Ophthalmology, The First Affiliated Hospital of Guangzhou Medical University, #151, Yanjiang West Road, Yuexiu District, Guangzhou, Guangdong 510120 China; 2 Department of Ophthalmology, Zhuzhou Hospital Affiliated to Xiangya School of Medicine, Central South University, #116, Changjiang South Road, Zhuzhou, Hunan 412000 China; 3 State Key Laboratory of Ophthalmology, Zhongshan Ophthalmic Center, Sun Yat-Sen University, Guangdong Provincial Key Laboratory of Ophthalmology and Visual Science, Guangdong Provincial Clinical Research Center for Ocular Diseases, Guangzhou, 510060 China; 4 Eye Center of Xiangya Hospital, Hunan Key Laboratory of Ophthalmology, Central South University, Changsha, 410008 China; 5 Department of Ophthalmology, Guangzhou Red Cross Hospital, Jinan University, Guangzhou, 510240 China

**Keywords:** Optical metrics, Spearman rank correlation, Intraocular lenses, Comparison

## Abstract

**Background:**

Postoperative performance, including best corrected distance visual acuity (BCDVA) and optical metrics (from the OQAS and iTrace devices), was compared among 4 different intraocular lenses (IOLs).

**Methods:**

This prospective observational study included 104 eyes from 104 subjects who underwent cataract surgery combined with implantation of 4 different IOLs: monofocal (Mon) IOLs, segmental refractive (SegRef) IOLs, diffractive (Dif) IOLs and extended depth of focus (EDoF) IOLs. Postoperative BCDVA and optical metrics were collected at the 6th month. The OQAS optical metrics included the objective scattering index (OSI), Strehl ratio (SR), modulation transfer function (MTF) cut-off frequency, and predicted visual acuity (PVA); the iTrace optical metrics included blur/double vision, glare/halo, starburst, mixed focus, night myopia, and night hyperopia.

**Results:**

There was no significant difference in BCDVA among the 4 groups (*P* = 0.059; power = 70.3%). Differences were observed in all OQAS optical metrics among the groups (all *P* < 0.001). Overall, Mon IOLs and EDoF IOLs exhibited better performance than Dif IOLs and SegRef IOLs. Starburst was the only iTrace optical metric that differed among the groups (*P* < 0.001): SegRef IOLs caused more starbursts than Mon IOLs (*P* = 0.001), Dif IOLs (*P* = 0.006) and EDoF IOLs (*P* < 0.001). Spearman rank correlation analysis was used to determine the relationships among the iTrace optical metrics, OQAS optical metrics and BCDVA: starburst was negatively correlated with BCDVA, PVA at contrasts of 100% and 20%, OSI, and MTF cut-off frequency (all *P* ≤ 0.001); mixed focus was positively correlated with BCDVA, PVA at contrasts of 100% and 20%, OSI, and MTF cut-off frequency (all *P* ≤ 0.001).

**Conclusions:**

Postoperative BCDVA and optical metrics varied among the different IOLs, which should be taken into account in the selection and management of IOLs for cataract patients.

**Trial registration:**

This study was approved by the First Affiliated Hospital of Guangzhou Medical University Ethical Review Board (No. 50 2022).

## Abstract (de)

**What was known:**

1. EDoF IOLs, one type of presbyopia-correcting IOL, provide excellent distance visual acuity and optical quality for patients undergoing cataract surgery.
2. Among patients undergoing cataract surgery, the use of presbyopia-correcting IOLs could cause more subjective photic phenomena than the use of monofocal IOLs.

**What this paper adds:**

1. The results of this prospective observational study demonstrated that EDoF IOLs exhibited better postoperative performance than SegRef IOLs and Dif IOLs.
2. Certain presbyopia-correcting IOLs provide VA at different distances by slitting light and creating additional foci, which inevitably results in a compromise in optical quality. These considerations should be considered in IOL selection.

## Introduction

Approximately 95 million people worldwide are estimated to suffer from cataracts. In low- and middle-income countries, cataracts remain the leading cause of blindness [1]. In recent decades, we have witnessed an increase in visual demands among the cataract population. This trend could be attributed to advancements in surgical techniques and intraocular lenses (IOLs) [1–5]. Currently, in addition to traditional monofocal IOLs (Mon IOLs), presbyopia-correcting IOLs, such as segmental refractive IOLs (SegRef IOLs), diffractive IOLs (Dif IOLs), and extended depth of focus IOLs (EDoF IOLs), are widely used.

However, with the increasing use of presbyopia-correcting IOLs, the incidence of undesirable photic phenomena after cataract surgery has also increased [6, 7]. The variety of IOL used somehow determines the postoperative visual performance in patients [8]. Some studies have reported a lower intensity of photic phenomena with EDoF IOLs than with other presbyopia-correcting IOLs [9–11], with slight “halo”, “starburst”, and “glare” effects [12]. Various photic phenomena hinder ophthalmologists from accurately assessing patients’ visual states. Therefore, there is a growing demand for improved methods to assess the visual status of patients.

In this study, optical metrics from the iTrace device, including blur/double vision, glare/halo, starburst, mixed focus, night myopia, and night hyperopia, were utilized to simulate the visual situation of patients. Furthermore, best corrected distance visual acuity (BCDVA) and optical metrics from the OQAS device, including the objective scattering index (OSI), Strehl ratio (SR), modulation transfer function (MTF) cut-off frequency, and predicted visual acuity (PVA), were collected postoperatively. We aimed to investigate the postoperative performance of Mon IOLs, SegRef IOLs, Dif IOLs and EDoF IOLs and to explore the association among the iTrace optical metrics, OQAS optical metrics, and BCDVA.

## Methods

### Study sample

This prospective observational study included 104 eyes from 104 subjects who underwent cataract surgery combined with implantation of IOLs at the First Affiliated Hospital of Guangzhou Medical University, Guangzhou, China, from May 2022 to May 2023. Subjects selected the IOL according to their preferences, and were divided into 4 groups: Mon IOLs, 24 subjects; SegRef IOLs, 25 subjects; Dif IOLs, 29 subjects; and EDoF IOLs, 26 subjects. The inclusion criteria included age ranging from 50 to 80 years, axial length ranging from 21.0 mm to 26.0 mm, corneal astigmatism less than 1.0 D, corneal endothelial cell density greater than 2000/mm^2^, and no pupillary abnormalities. Subjects with a history of severe dry eye, corneal pathologies, glaucoma, uveitis, retinal abnormalities, ocular trauma, previous corneal or intraocular surgery, high myopia, or connective tissue disease were excluded from the study. All participants signed informed consent prior to the study. The study followed the Declaration of Helsinki tenets of 1975 and received approval from the First Affiliated Hospital of Guangzhou Medical University Ethical Review Board (No. 50 2022).

### Intraocular lens

The NS-60YG (Nidek Co. Ltd., Japan) is a modified C-loop Mon IOL with an aspheric optical side manufactured from hydrophobic acrylic. The total diameter of the IOLs is 13.0 mm, with an optic size of 6.0 mm [13].

The SBL-3 (Lenstec, Christ Church, Barbados) is a SegRef IOL manufactured from hydrophilic acrylic material with two distinct zones. One zone is for distance vision, and the other is for near vision, with a near addition of + 3.0 D (+ 2.5 D on the spectacle plane) in the inferior anterior optic. The distance zone is separated from the near addition zone by using a small wedge-shaped transition zone. The total diameter of the multifocal IOLs is 11.0 mm, with an optic size of 5.75 mm [14].

Tecnis Symfony ZXR00 (Johnson & Johnson Vision, Santa Ana, California, USA) is an EDoF IOL with a biconvex hydrophobic UV-filtering C-loop manufactured from hydrophilic acrylic material. It has a negative spherical aberration of 0.27 μm on the anterior surface. To expand the field of vision, the posterior surface has an achromatic design and an echelette, which is a type of diffraction grating. Within the 9 rings of the diffractive zone, the refractive area has a diameter of 1.7 mm. The total diameter of the multifocal IOLs is 13.0 mm, with an optic diameter of 6.0 mm [15, 16].

The Tecnis ZMB00 (Johnson & Johnson Vision, Santa Ana, California, USA) is a Dif IOL that uses a material and structure similar to that of ZXR00. The main difference between these two IOLs is that the back surface of ZMB00 consists of 22 concentric diffractive rings with a near addition of + 4.0 D (+ 3.0 D on the spectacle plane). The diffractive zone has a refractive area of 1.0 mm in diameter and a 1:1 distribution between two foci [16, 17].

### Assessment

Preoperative assessment: All subjects underwent a standardized ophthalmic examination, including preoperative uncorrected visual acuity (UCVA), manifest refraction, intraocular pressure (IOP), corneal topography (Pentacam, Oculus, Wetzlar, Germany), endothelial cell count (SP 2000P specular microscope, Topcon, Norway, Europe BV), slit-lamp examination (SL115; Carl Zeiss, Oberkochen, Germany), dilated fundus examination, and retinal optical coherence tomography (OCT, Carl Zeiss Meditec AG, Jena, Germany). In addition, the axial length (AL), anterior chamber depth (ACD), and corneal curvature were measured by an IOL Master (Carl Zeiss Meditec AG, Jena, Germany).

**Fig. 1:**
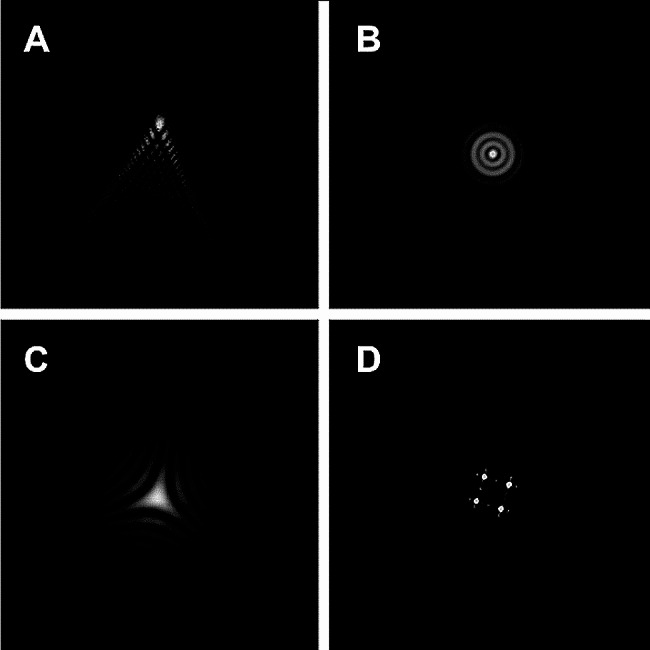
The simulative morphology of the point spread function (PSF) when wavefront aberration presented in the iTrace device. (**A**): blur/double vision; (**B**): glare/halo; (**C**): starburst; (**D**): mixed focus

**Fig. 2:**
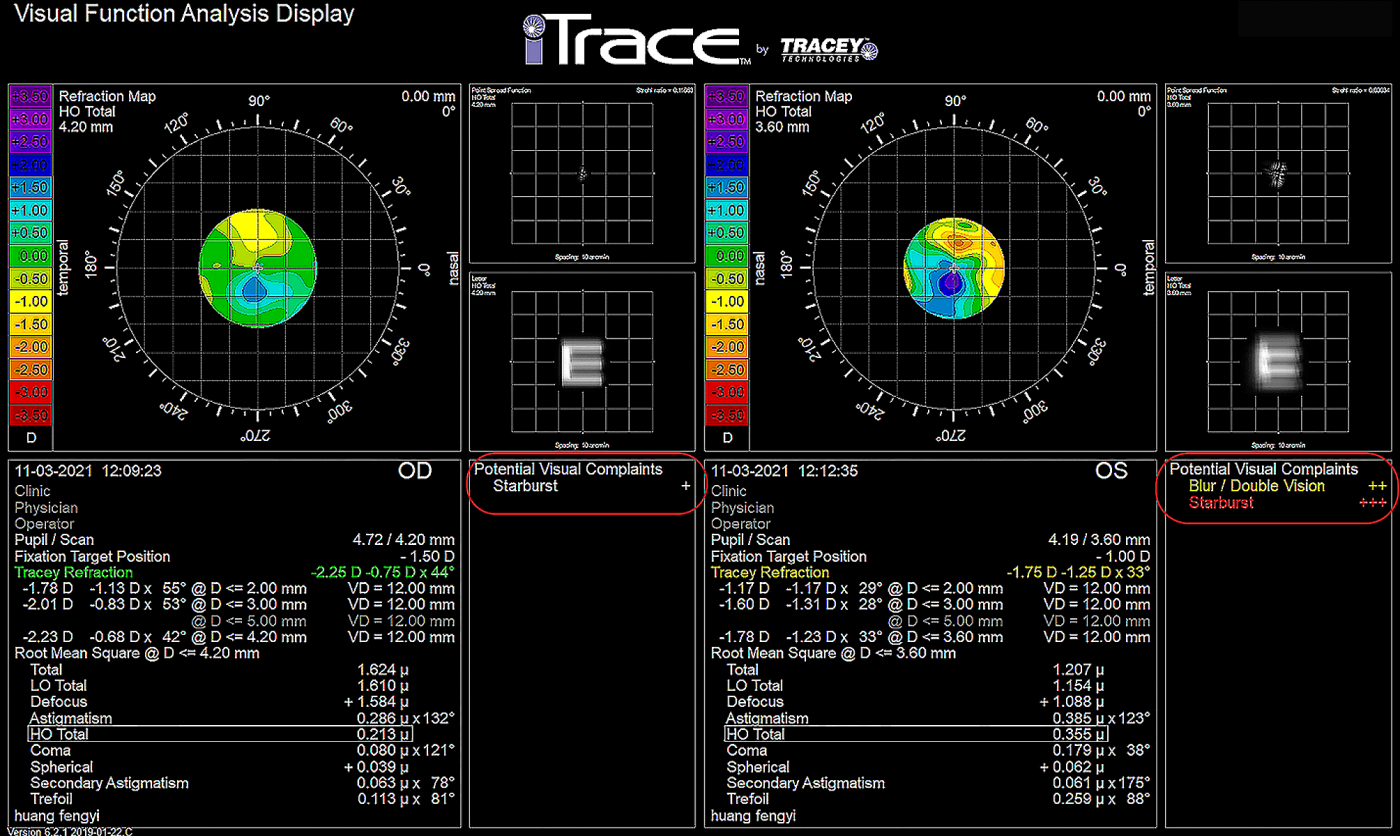
Optical metrics collected through the iTrace device. (red frames)

Postoperative assessment: BCDVA and optical metrics were collected 6 months after surgery. BCDVA was evaluated using logarithm of the minimum angle of resolution (logMAR) charts at a distance of 5 m. An OQAS II device (Visiometrics, Terrassa, Spain) was used to collect OQAS optical metrics, including the objective scattering index (OSI), Strehl ratio (SR), modulation transfer function (MTF) cut-off frequency (c/deg), and predicted visual acuity (PVA) at contrasts of 100%, 20%, and 9%. Before statistical analysis, all PVA data were converted into logMAR format. An iTrace device (Tracey Technology, Houston, Texas) was used to collect the wavefront aberrations [18, 19] and iTrace optical metrics of the subjects. The iTrace optical metrics included blur/double vision, glare/halo, starburst, mixed focus, night myopia, and night hyperopia. Through its built-in calculation formula, the iTrace device simulated the morphology of the point spread function (PSF) (Fig. 1) and presented the type and degree of the iTrace optical metrics (Fig. 2) when wavefront aberrations occurred. For instance, when a coma aberration occurred alone, a comet tail appeared in the image, which was described as “blur” or “double vision” (Fig. 1A). When a spherical aberration occurred alone, concentric circles appeared in the image, which were described as “glare” or “halo” (Fig. 1B). When a trefoil aberration occurred alone, the image seemed to be a star, which was described as “starburst” (Fig. 1C). When a second-order astigmatism aberration occurred alone, multiple focal points appeared in the image, which was described as “mixed focus” (Fig. 1D). The severity of the iTrace optical metrics was classified into four grades: none (-), mild (+), moderate (++), and severe (+++). All the examinations were completed by the same technicians.

### Surgical procedure

All surgeries were performed by the same experienced surgeon under topical anaesthesia. The Centurion Vision System (Alcon, Fort Worth, TX, USA) was used to obtain a clear corneal phacoemulsification through a 2.2 mm main incision and a 1 mm lateral incision in all subjects. The Verion Image Guided System (Alcon, Fort Worth, TX, USA) was used to demonstrate a capsulorhexis diameter of 5.0 mm and the centre of the IOLs.

### Statistical analysis

The Kolmogorov‒Smirnov test was applied to assess the normality of the data. Numbers are presented as counts (percentage) for categorical variables, mean (standard deviation, SD) for normally distributed continuous variables, and median (interquartile range, IQR) for nonnormally distributed continuous variables. Comparisons between groups of categorical variables were performed using a chi-square test or Fisher’s exact test. Normally distributed continuous data were compared by analysis of variance (ANOVA): the least significant difference (LSD) t test was applied when the assumption of homogeneity of variance was satisfied; otherwise, Tamhane’s T2 test was used. Nonnormally distributed continuous data and grade data were compared by the Kruskal‒Wallis H test, and multiple comparisons were performed using the Bonferroni correction. To evaluate the associations among the iTrace optical metrics, OQAS optical metrics and BCDVA, Spearman rank correlation tests were performed. The power of postoperative comparison analysis among groups and correlation analyses were tested using PASS software 2021 (NCSS, Kaysville, UT, USA), and a value of power above 80% was considered credible. *P* < 0.05 was considered to indicate statistical significance. SPSS Statistics v26.0 (IBM, Chicago, IL) was used for all the statistical analyses.

## Results

### Baseline analysis

A total of 104 eyes from 104 subjects were analysed. There were no statistically significant differences among the groups in terms of the preoperative ratio of right to left eyes, sex, age, IOP, UCVA, or AL (all *P* > 0.05) (Table 1).

**Table 1:** Baseline analysis among the 4 groups

	Total	Mon	SegRef	Dif	EDoF	x² / K	P
Eyes(right/left)	55/49	12/12	16/9	13/16	14/12	2.085	0.56
Gender(male/female)	42/62	10/14	13/12	7/22	12/14	4.956	0.18
Age (year)^a^	68.7 (8.2)	67.8 (8.2)	69.6 (9.1)	68.4 (8.8)	69.2 (6.4)	1.968	0.58
IOP (mmHg)^a^	13.7 (2.5)	13.2 (2.5)	14.2 (2.49)	13.6 (2.5)	13.7 (2.46)	1.482	0.69
UCVA (logMAR)^b^	0.40 (0.56)	0.56 (0.68)	0.40 (0.36)	0.36 (0.51)	0.40 (0.66)	4.032	0.26
AL (mm)^b^	23.39 (1.12)	23.24 (1.27)	23.46 (0.84)	23.73 (1.21)	23.31 (1.41)	4.116	0.25

^a^Normally distributed continuous data were described by mean (standard deviation)

^b^Nonnormally distributed continuous data were described as the median (interquartile range)

Mon = monofocal IOLs; SegRef = segmental refractive IOLs; Dif = diffractive IOLs; EDoF = extended depth of focus IOLs; UCVA = uncorrected visual acuity; IOP = intraocular pressure; AL = axial lengths

UCVA was shown in logMAR (logarithm of the minimum angle of resolution)

*P < 0.05, **P ≤ 0.01; ***P ≤ 0.001

### BCDVA

**Fig. 3:**
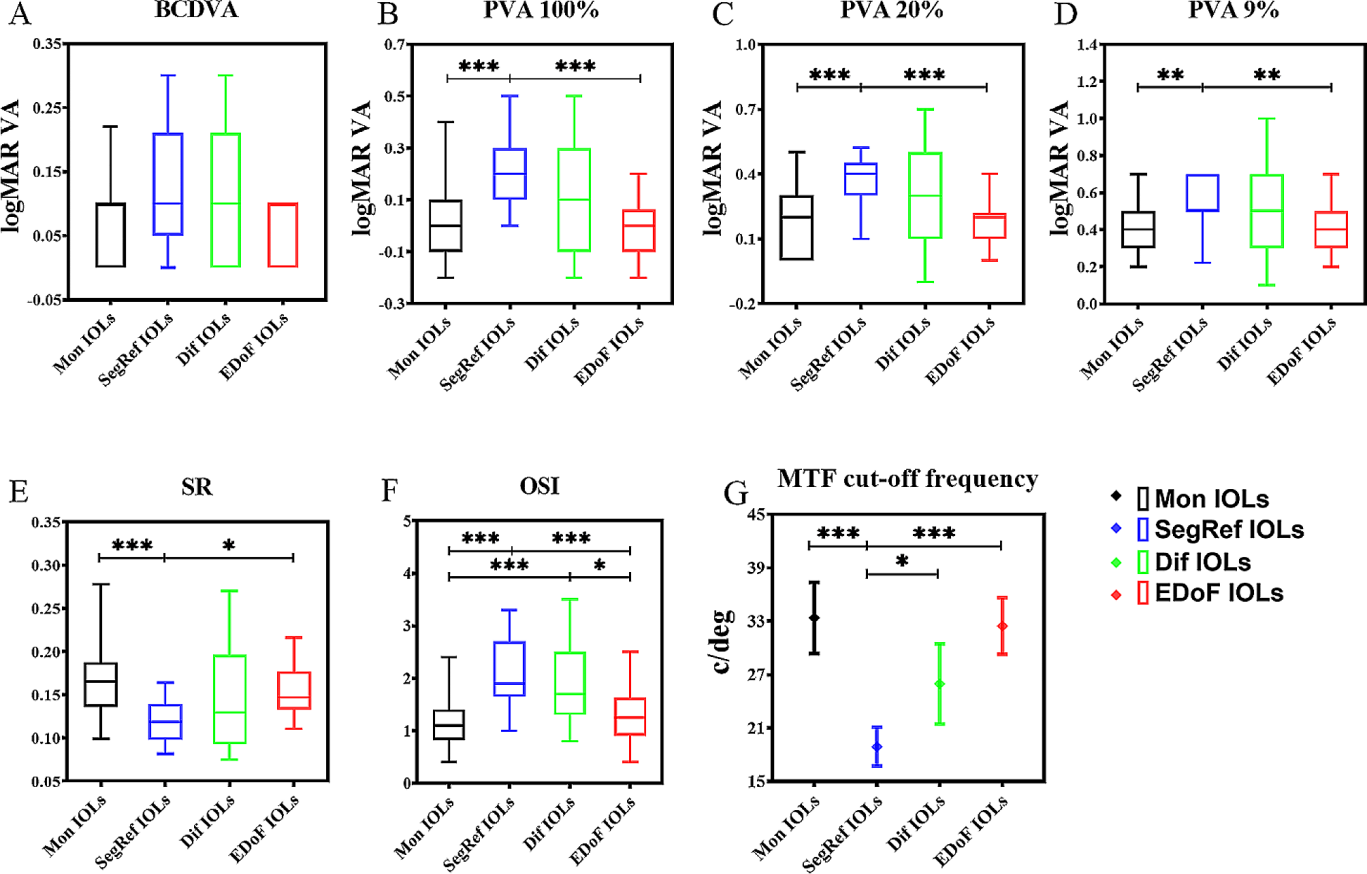
Comparisons of postoperative BCDVA and the OQAS optical metrics among the 4 groups. (**A**): Postoperative best corrected distance visual acuity (BCDVA). (**B**): Postoperative predicted visual acuity (PVA) at contrasts of 100%. (**C**): Postoperative PVA at contrasts of 20%. (**D**): Postoperative PVA at contrasts of 9%. (**E**): Postoperative strehl ratio (SR). (**F**): Postoperative objective scattering index (OSI). (**G**): Postoperative modulation transfer function (MTF) cut-off frequency. A, B, C, D, E, and F were presented in box and whiskers plots as they were nonnormally distributed; G was presented in plots with mean and error bar as it was normally distributed. *P < 0.05, **P ≤ 0.01; ***P ≤ 0.001

There was no statistically significant difference in BCDVA among the 4 groups at 6 months after surgery (*P* = 0.059; power = 70.3%) (Table 2) (Fig. 3A).

**Table 2:** BCDVA and the OQAS optical metrics at 6th months postoperatively among the 4 groups

	Mon	SegRef	Dif	EDoF	K/F	P	power
BCDVA^a^	0.10 (0.10)	0.10 (0.16)	0.10 (0.21)	0.10 (0.10)	7.439	0.059	70.3%
PVA 100%^a^	-0.04 (0.21)	0.22 (0.2)	0.10 (0.38)	0 (0.16)	30.962	< 0.001***	99.9%
PVA 20%^a^	0.15 (0.30)	0.40 (0.18)	0.30 (0.42)	0.15 (0.13)	24.910	< 0.001***	99.9%
PVA 9%^a^	0.40 (0.22)	0.52 (0.18)	0.52 (0.40)	0.40 (0.22)	17.537	0.001***	99.8%
SR^a^	0.17 (0.05)	0.12 (0.04)	0.13 (0.10)	0.15 (0.04)	18.812	< 0.001***	99.9%
OSI^a^	1.10 (0.60)	1.90 (1.10)	1.70 (1.20)	1.25 (0.70)	32.257	< 0.001***	99.9%
MTF cut-off frequency (c/deg)^b^	33.38 (9.49)	18.9 (5.29)	25.97 (11.82)	32.47 (7.85)	13.782	< 0.001***	99.9%

^a^Nonnormally distributed continuous data were described as the median (interquartile range)

^b^Normally distributed continuous data were described by mean (standard deviation)

Mon = monofocal IOLs; SegRef = segmental refractive IOLs; Dif = diffractive IOLs; EDoF = extended depth of focus IOLs; BCDVA = best corrected distance visual acuity; PVA 100% = predicted visual acuity at contrast of 100%; PVA 20% = predicted visual acuity at contrast of 20%; PVA 9% = predicted visual acuity at contrast of 9%; SR = strehl ratio; MTF = Modulation transfer function; OSI = objective scattering index

The OQAS optical metrics include PVA 100%, PVA 20%, PVA 9%, SR, MTF cutoff frequency, and OSI

BCDVA and PVA were shown in logMAR (logarithm of the minimum angle of resolution)

*P < 0.05, **P ≤ 0.01; ***P ≤ 0.001

### Optical metrics

Significant differences among the groups were found in all of the postoperative OQAS optical metrics, including PVA 100%, PVA 20%, PVA 9%, SR, OSI, and MTF cut-off frequency (all *P* < 0.001) (Table 2). According to post hoc testing and multiple comparisons (Table 3), Mon IOLs and EDoF IOLs showed similar performances in terms of OQAS optical metrics (all *P* > 0.05) (Fig. 3). Furthermore, Dif IOLs and SegRef IOLs exhibited comparable performances (all *P* > 0.05), except for the MTF cut-off frequency (*P* = 0.005) (Fig. 3). Overall, the OQAS optical metrics of the EDoF and Mon IOLs were better than those of the Dif and SegRef IOLs.

**Table 3:** Post hoc testing and multiple comparisons analysis of BCDVA and the OQAS optical metrics

	Mon vs. SegRef				Mon vs. Dif					Mon vs. EDof					SegRef vs. Dif					SegRef vs. EDof					Dif vs. EDof					Total	
	Test statistic		P	Test statistic			P	Test statistic				P	Test statistic				P	Test statistic				P	Test statistic				P			K/F	P
BCDVA^a^	There was no statistical difference among the four groups, and no multiple comparisons results were outputted from the SPSS software																													7.439	0.059
PVA 100%^a^	-39.979	**< 0.001** ***			-19.669	0.105				0.463	1.000				20.310	0.079				40.442	**< 0.001** ***				20.132	0.078				30.962	**< 0.001** ***
PVA 20%^a^	-35.517	**< 0.001** ***			-19.455	0.111				0.101	1.000				16.063	0.295				35.618	**< 0.001** ***				19.556	0.093				24.910	**< 0.001** ***
PVA 9%^a^	-26.878	**0.009** **			-18.200	0.152				2.311	1.000				8.679	1.000				29.189	**0.002** **				20.511	0.060				17.537	**0.001** ***
SR^a^	34.660	**< 0.001** ***			21.534	0.058				8.654	1.000				-13.126	0.665				-26.006	**0.012** *				-12.881	0.683				18.812	**< 0.001** ***
OSI^a^	-41.142	**< 0.001** ***			-31.976	**0.001** ***				-7.024	1.000				9.166	1.000				34.118	**< 0.001** ***				24.952	**0.013** *				32.257	**< 0.001** ***
MTF cut-off frequency^b^	14.478	**< 0.001** ***			7.411	0.084				0.904	0.999				-7.067	**0.036** *				-13.573	**< 0.001** ***				-6.507	0.108				13.782	**< 0.001** ***

^a^Nonnormally distributed data were compared by the Kruskal‒Wallis H test, and multiple comparisons were performed using the Bonferroni correction

^b^Normally distributed data were compared by analysis of variance (ANOVA): the least significant difference (LSD) t test was applied when the assumption of homogeneity of variance was satisfied; otherwise, Tamhane’s T2 test was used

Mon = monofocal IOLs; SegRef = segmental refractive IOLs; Dif = diffractive IOLs; EDoF = extended depth of focus IOLs; BCDVA = best corrected distance visual acuity; PVA 100% = predicted visual acuity at contrast of 100%; PVA 20% = predicted visual acuity at contrast of 20%; PVA 9% = predicted visual acuity at contrast of 9%; SR = strehl ratio; MTF = Modulation transfer function; OSI = objective scattering index

*P < 0.05, **P ≤ 0.01; ***P ≤ 0.001

The postoperative iTrace optical metrics of the 4 groups were analysed (Table 4). There were no significant differences among the groups in terms of blur/double vision, glare/halo, mixed focus, night myopia or night hyperopia (all *P* > 0.05) at 6 months after surgery, but the difference in starburst was significant (*P* < 0.001). Multiple comparisons revealed that the incidence of starburst was greater for SegRef IOLs than for Mon IOLs (*P* = 0.001), Dif IOLs (*P* = 0.006), and EDoF IOLs (*P* < 0.001).

**Table 4:** The iTrace optical metrics at the 6th months postoperatively among the 4 groups

	Mon	SegRef	Dif	EDoF	Total	P	power
blur/double vision ^a^						0.79	99.9%
0	15 (21.4%)	16 (22.9%)	21 (30.0%)	18 (25.7%)	70 (100%)		
+	7 (23.3%)	8 (26.7%)	8 (26.7%)	7 (23,3%)	30 (100%)		
++	2 (66.7%)	0 (0%)	0 (0%)	1 (33.3%)	3 (100%)		
+++	0 (0%)	1 (100%)	0 (0%)	0 (0%)	1 (100%)		
glare/halo ^a^						0.70	99.9%
0	21 (24.4%)	21 (22.4%)	22 (25.6%)	22 (25.6%)	86 (100%)		
+	3 (16.7%)	4 (22.2%)	7 (38,9%)	4 (22.2%)	18 (100%)		
++	0 (0%)	0 (0%)	0 (0%)	0 (0%)	0 (0%)		
+++	0 (0%)	0 (0%)	0 (0%)	0 (0%)	0 (0%)		
starburst ^a^						< 0.001***	99.5%
0	9 (24.3%)	3 (8.1%)	10 (27.0%)	15 (40.5%)	37 (100%)		
+	12 (32.4%)	7 (18.9%)	11 (29.7%)	7 (18.9%)	37 (100%)		
++	3 (13.0%)	9 (39.1%)	8 (34.8%)	3 (13.0%)	23 (100%)		
+++	0 (0%)	6 (85.7%)	0 (0%)	1 (14.3%)	7 (100%)		
mixed focus ^a^						0.84	59.5%
0	16 (23.5%)	17 (25.0%)	20 (29.4%)	15 (22.1%)	68 (100%)		
+	8 (22.9%)	7 (20.0%)	9 (25.7%)	11 (31.4%)	35 (100%)		
++	0 (0%)	1 (100%)	0 (0%)	0 (0%)	1 (100%)		
+++	0 (0%)	0 (0%)	0 (0%)	0 (0%)	0 (0%)		
night myopia ^a^						0.43	99.9%
0	22 (23.7%)	22 (23.7%)	24 (25.8%)	25 (26.9%)	93 (100%)		
+	2 (18.2%)	3 (27.3%)	5 (45.5%)	1 (9.1%)	11(100%)		
++	0 (0%)	0 (0%)	0 (0%)	0 (0%)	0 (0%)		
+++	0 (0%)	0 (0%)	0 (0%)	0 (0%)	0 (0%)		
night hyperopia ^a^						0.96	99.9%
0	21 (23.1%)	22 (24.2%)	26 (28.6%)	22 (24.2%)	91 (100%)		
+	3 (23.1%)	3 (23.1%)	3 (23.1%)	4 (30.8%)	13 (100%)		
++	0 (0%)	0 (0%)	0 (0%)	0 (0%)	0 (0%)		
+++	0 (0%)	0 (0%)	0 (0%)	0 (0%)	0 (0%)		

^a^ Categorical variables were presented as counts (percentage)

Mon = monofocal IOLs; SegRef = segmental refractive IOLs; Dif = diffractive IOLs; EDoF = extended depth of focus IOLs

The iTrace optical metrics include blur/double vision, glare/halo, starburst, mixed focus, night myopia, and night hyperopia

*P < 0.05, **P ≤ 0.01; ***P ≤ 0.001

Results of multiple comparisons were presented as follows: Starburst: SegRef IOLs caused more cases of starburst than Mon IOLs (*P* = 0.001), Dif IOLs (*P* = 0.006), and EDoF IOLs (*P* < 0.001). No statistical differences between groups in blur/double vision, glare/halo, mixed focus, night myopia or night hyperopia (all *P* > 0.05)

### Spearman rank correlation analysis

Spearman rank correlation analysis was used to investigate the relationships among the iTrace optical metrics, OQAS optical metrics and BCDVA. Starburst and mixed focus were the only two iTrace optical metrics that exhibited statistical correlation with the OQAS optical metrics and BCDVA (Table 5).

**Table 5:** Spearman rank correlation analysis of the relationships among the iTrace optical metrics, OQAS optical metrics and BCDVA. (*N* = 104)

	Sample size	Starbursts ^b^				Mixed focus ^b^			
		Correlation coefficient	P	power		Correlation coefficient	P	power	
BCDVA	104	0.427	< 0.001***	98.8%		-0.362	< 0.001***	96.4%	
PVA 100% ^a^	104	0.371	< 0.001***	96.1%		-0.366	< 0.001***	96.2%	
PVA 20% ^a^	104	0.328	< 0.001***	93.5%		-0.328	< 0.001***	83.8%	
PVA 9% ^a^	104	0.163	0.098	37.3%		-0.144	0.146	28.1%	
SR ^a^	104	-0.208	0.034*	51.1%		0.09	0.366	16.1%	
OSI ^a^	104	0.442	< 0.001***	99.0%		-0.342	< 0.001***	93.0%	
MTF cut-off frequency ^a^	104	-0.365	0.001***	95.5%		0.356	0.001***	95.4%	

^a^ The OQAS optical metrics include PVA 100%, PVA 20%, PVA 9%, SR, OSI and MTF cutoff frequency

^b^ The iTrace optical metrics include blur/double vision, glare/halo, starburst, mixed focus, night myopia, and night hyperopia. Starburst and mixed focus were the only two iTrace optical metrics that exhibited statistical correlation with the OQAS optical metrics and BCDVA

BCDVA = best corrected distance visual acuity; PVA 100% = predicted visual acuity at contrast of 100%; PVA 20% = predicted visual acuity at contrast of 20%; PVA 9% = predicted visual acuity at contrast of 9%; SR = strehl ratio; MTF = Modulation transfer function; OSI = objective scattering index

*P < 0.05, **P ≤ 0.01; ***P ≤ 0.001

Starburst was negatively associated with BCDVA, PVA 100%, PVA 20%, OSI, and the MTF cut-off frequency (all *P* ≤ 0.001). In contrast, mixed focus was positively associated with postoperative BCDVA, PVA 100%, PVA 20%, OSI, and MTF cut-off frequency (all *P* ≤ 0.001).

## Discussion

Presbyopia-correcting IOLs are becoming more popular for meeting the vision demands of modern life. In this study, the optical metrics of eyes implanted with presbyopia-correcting IOLs were a focus of the visual evaluations and provide a reference for technical improvements. The OQAS II device evaluates optical performance by measuring the image formed on the retina, combining optical aberrations and ocular media transparency [20–22]. Additionally, the postoperative photic phenomenon serves as another indicator for evaluating the performance of IOLs [7, 23]. The iTrace device simulates potential photic phenomena that may occur in patients who have undergone cataract and IOL implantation surgery. In the present study, optical metrics and BCDVA were collected to evaluate the postoperative performance of different types of IOLs.

The BCDVA and optical metrics collected through the OQAS were important indicators for postoperative follow-up of eyes implanted with IOLs, providing a possible path for ophthalmologists to investigate postoperative performance [24]. In the present study, while no significant difference was observed in BCDVA, there were variations in the OQAS optical metrics at 6 months after surgery among the groups (Table 2). Overall, the EDoF IOLs and Mol IOLs showed better performance in PVA 100%, PVA 20%, PVA 9%, SR, ISO, and MTF cut-off frequencies (Fig. 3), which was consistent with previous studies [16, 25, 26]. In addition, there was no significant difference in the other OQAS optical metrics between the SegRef IOL group and Dif IOL group, except for the MTF cut-off frequency (Fig. 3). A previous study reported that Dif IOLs appear to be comparable to SegRef IOLs in terms of contrast sensitivity [27]. The differences in OQAS optical metrics among groups can be attributed to the unique optical designs of IOLs for light splitting. Simultaneous vision requires sufficient energy to be distributed to two or more foci [23, 28]. Common presbyopia-correcting IOLs, such as SegRef IOLs and Dif IOLs, split light into multiple foci and disperse light energy, which may induce a slight optical interference [29]. In contrast, EDoF IOLs spread light across a range and provide a continuous range of vision without causing a slight reduction in OQAS optical metrics [25, 27]. BCDVA (power = 70.3%) was the only postoperative indicator that did not achieve the target power (80%) (Table 2). Therefore, considering the significant differences in OQAS optical metrics among the groups, we could not rule out the possibility of differences in BCDVA among the groups, although the *P* value (0.059) was marginally greater than the critical value (0.05). Studies with larger sample sizes are required to detect a difference in BCDVA among groups.

In addition to BCDVA and optical metrics collected through OQAS, the other optical metrics collected through iTrace provided an additional dimension for evaluating the visual status of patients—photic phenomena. Photic phenomena are a common cause of decreased satisfaction among patients who underwent implantation of presbyopia-correcting IOLs, even those with excellent VA [30]. Researchers in previous studies tended to collect information about photic phenomena through questionnaires [10, 11, 26, 29]. Although the patient comments provided by questionnaires may be a proactive approach, they could be influenced by biases arising from different populations, educational levels, or even emotions [31, 32]. On the other hand, the iTrace optical metrics collected through the AI system are more intuitive and stable. Since the patients in the present study were from different social backgrounds, we introduced iTrace optical metrics into the visual evaluation system to avoid subjective unreliability, which has rarely been reported before.

Among the iTrace optical metrics, starburst was found to be the only photic phenomenon that varied between groups (Table 4). Although it has been reported that presbyopia-correcting IOLs may cause more photic disturbances [6, 7, 26, 28], there were no statistically significant differences in other iTrace optical metrics between the Mon IOL group and the other groups (Table 4). This result differed from that of a previous study [29] that reported similar incidence rates of “halo” and “glare” between the SegRef IOLs and Dif IOLs. This difference could be attributed to variations in indicator collection approaches and tested cohorts. We hypothesized that the unique optical surface design of the SegRef IOLs might also cause this phenomenon. On the other hand, the iTrace optical metrics exhibited similar results for both Dif IOLs and EDoF IOLs, which was consistent with previous studies. No significant difference in the occurrence or intensity of “glare” or “halos” was observed between these two types of IOLs [28]. Moreover, a specific subscale of the NEI-RQL instrument revealed comparable frequencies of “glare” in both types of IOLs [26]. This similarity could be attributed to the hydrophilic acrylic material and biconvex hydrophobic UV-filtering C-loop of the Dif IOLs and EDoF IOLs. However, it is worth noting that the definitions of various photic phenomena in previous studies partially differed from the iTrace optical metrics used in our study. For example, “glare” in the NEI-RQL instrument scale includes “starburst”, “halo” and “glare” [33]. In this study, the iTrace optical metrics used propose novel concepts for future photic phenomena studies.

Spearman rank correlation analysis (Table 5) revealed that starburst tended to have a statistically significant correlation with worse BCDVA, PVA 100%, PVA 20%, OSI, and the MTF cut-off frequency. Although there was a significant correlation between the SR and starburst (*P* < 0.05), given the relatively low correlation coefficient (-0.208), we could not draw a definitive conclusion regarding their connection. Interestingly, mixed focus had the opposite relationship. The eyes with mixed focus were more likely to have better BCDVA, PVA 100%, PVA 20%, OSI, and MTF cut-off frequencies. The paradox of this difference is worth exploring.

VA is commonly used to evaluate patient prognosis after cataract surgery. However, visual status can sometimes be contradictory to the occurrence of the presented indicators. Photic phenomena may be a reason why some patients complain about their vision even though the VA test results are good [30]. The response of photoreceptors to the input signal is graded according to the capture of photons within the photoreceptor [34]. Although spatial information is lost when only one photoreceptor is stimulated, images of the letter E must be distributed over a sufficient number of photoreceptors to be recognized. Mixed focus is a unique optical interference that scatters light into a quaternion quadrant, casting four duplicated clear spots without causing distortion (Fig. 1C). Based on these optical characteristics, we hypothesize that mixed focus reflects light in a regular form and increases the receiving area of the retina. However, the other iTrace optical metrics distort the flare so that the image becomes blurry (Fig. 1). In addition, some types of higher-order aberrations (particularly spherical aberrations, coma, and second-order astigmatism) could improve the depth of focus (DoF) [35]. Thus, mixed focus, associated with second-order astigmatism, could maintain a better DoF, resulting in higher OQAS optical metrics. However, another study reached the opposite conclusion that removing second-order astigmatism could improve image quality and increase the DoF [36]. This difference could result from the use of different test cohorts. Moreover, the influence of mixed focus on visual status should be further studied in the future.

## Conclusion

The present study demonstrated the different characteristics of Mon IOLs, Dif IOLs, SegRef IOLs, and EDoF IOLs in terms of optical metrics and BCDVA. In this study, there was no statistically significant difference in BCDVA among the four groups at 6 months after surgery. Moreover, we observed that Mon IOLs and EDoF IOLs had better OQAS optical metrics and fewer starburst effects postoperatively; Dif IOLs seemed to perform moderately among the four IOLs; and SegRef IOLs showed slightly poorer OQAS optical metrics and more starburst effects after surgery compared with the other groups. Therefore, for patients with greater driving demands, especially at night, SegRef IOLs should be selected with caution to reduce secondary outcomes of IOLs, such as car accidents [30]. The associations between postoperative optical metrics (iTrace and OQAS) and BCDVA were illustrated; the OQAS optical metrics and BCDVA were negatively correlated with starburst but positively correlated with mixed focus. Nonparallelism among these measurement indicators was revealed in this study. Further investigation of optical metrics in patients with good BCDVA but low satisfaction is recommended to provide better personalized medical services.

## Data Availability

The data analyzed during the current study are available from the corresponding author on reasonable request.

## Abbreviations

IOLs: Intraocular lenses
Mon IOLs: Monofocal IOLs
SegRef IOLs: Segmental refractive IOLs
Dif IOLs: Diffractive IOLs
EDoF IOLs: Extended depth of focus IOLs
DoF: Depth of focus
VA: Visual acuity
UCVA: Uncorrected visual acuity
BCDVA: Best corrected distance visual acuity
IOP: Intraocular pressure
AL: Axial length
ACD: Anterior chamber depth
OSI: Objective scattering index
SR: Strehl ratio
MTF: Modulation transfer function
PVA: Predicted visual acuity
PSF: Point spread function
SD: Standard deviation
IQR: Interquartile range
logMAR: Logarithm of the minimum angle of resolution
